# Rationality for isobaric automorphic representations: the CM-case

**DOI:** 10.1007/s00605-018-1188-5

**Published:** 2018-05-21

**Authors:** Harald Grobner

**Affiliations:** Fakultät für Mathematik, University of Vienna, Oskar-Morgenstern-Platz 1, 1090 Vienna, Austria

**Keywords:** Period, *L*-function, Critical value, Isobaric sum, Cuspidal automorphic, 11F67 (Primary)11F70, 11G18, 11R39, 22E55 (Secondary)

## Abstract

In this note we prove a simultaneous extension of the author’s joint result with M. Harris for critical values of Rankin–Selberg *L*-functions $L(s,\Pi \times \Pi ')$ (Grobner and Harris in J Inst Math Jussieu 15:711–769, 2016, Thm. 3.9) to (i) general CM-fields *F* and (ii) cohomological automorphic representations $\Pi '=\Pi _1\boxplus \cdots \boxplus \Pi _k$ which are the isobaric sum of unitary cuspidal automorphic representations $\Pi _i$ of general linear groups of arbitrary rank over *F*. In this sense, the main result of these notes, cf. Theorem 1.9, is a generalization, as well as a complement, of the main results in Raghuram (Forum Math 28:457–489, 2016; Int Math Res Not 2:334–372, 2010. https://doi.org/10.1093/imrn/rnp127), and Mahnkopf (J Inst Math Jussieu 4:553–637, 2005).

## Rationality for isobaric automorphic representations: the general case

### Introductory comments: a leitfaden for the reader

The purpose of this note is to prove a broad generalization of our own rationality-result, [12, Thm. 3.9], established *ibidem* for critical values of the Rankin–Selberg *L*-function $L(s,\Pi \times \Pi ')$ of certain automorphic representations $\Pi \otimes \Pi '$ of $\mathrm{GL}_n\times \mathrm{GL}_{n-1}$ over an imaginary quadratic field $\mathcal {K}$. Our generalization of this result will be in terms of the nature of the base field $\mathcal {K}$, and even more importantly, of the nature of the automorphic representation $\Pi '$.

#### A short review of our result in [12]

To put ourselves *in medias res*, we will briefly recall our rationality-theorem, [12, Thm. 3.9]. It applies to a pair $(\Pi ,\Pi ')$ of a cohomological cuspidal automorphic representation $\Pi$ of $\mathrm{GL}_n(\mathbb {A}_\mathcal {K})$ and a cohomological abelian automorphic representation $\Pi '$ of $\mathrm{GL}_{n-1}(\mathbb {A}_\mathcal {K})$, i.e., an isobaric sum of distinct unitary Hecke characters $\Pi '=\chi \boxplus \cdots \boxplus \chi _{n-1}$, over imaginary quadratic fields $\mathcal {K}$. By a principle found in [14, 22, 27], which works in even greater generality as exploited in the latter references, one may attach a Whittaker period $p(\Pi )$ and $p(\Pi ')$ to such representations: Explained in due shortness, this period is defined by comparison of

(i) a fixed rational structure of the (unique) Whittaker model $W(\Pi _f)$ (resp. $W(\Pi '_f)$) of the finite part of the given automorphic representation and
(ii) a fixed rational structure on a (uniquely chosen) $\Pi _f$– (resp. $\Pi '_f$–) isotypic subspace in the cohomology $H^{b_n}(S_n,\mathcal {E}_\mu )$ (resp. $H^{b_{n-1}}(S_{n-1},\mathcal {E}_\lambda )$) of the adelic “locally symmetric space” $S_n$ (reps. $S_{n-1}$) in the lowest, possible degree $b_n$ (resp. $b_{n-1}$).

As both, the Whittaker model and the above cohomological model, are irreducible representations, their rational structures are unique up to multiplication by non-zero complex numbers. Hence, the Whittaker periods $p(\Pi )$ and $p(\Pi ')$ may simply be defined as a choice of normalization-factor, which makes the isomorphism between the Whittaker model and our cohomological model, induced from the global $\psi$-Fourier coefficient, respect the two fixed choices of rational structures on domain and target space.

Recall the Gauß-sum $\mathcal {G}(\omega _{\Pi '_f})$ of the central character $\omega _{\Pi '_f}$ of $\Pi '_f$ and assume that the coefficient modules $\mathcal {E}_\mu$ and $\mathcal {E}_\lambda$ in cohomology allow a non-trivial $\mathrm{GL}_{n-1}(\mathbb {C})$-equivariant intertwining $\mathcal {E}_\mu \otimes \mathcal {E}_\lambda \rightarrow \mathbb {C}$. Under these assumptions the rationality-theorem [12, Thm. 3.9] asserts that for every critical point of $L(s,\Pi \times \Pi ')$, i.e., for every half-integer $s_0=\tfrac{1}{2}+m$, for which the archimedean *L*-factors on both sides of the functional equation of $L(s,\Pi \times \Pi ')$ are holomorphic, there is a non-zero archimedean period $p(m,\Pi _\infty ,\Pi '_\infty )\in \mathbb {C}^*$, only depending on *m*, $\Pi _\infty$ and $\Pi '_\infty$, such that

1.1 $$\begin{aligned} L\left( \tfrac{1}{2}+m,\Pi _f \times \Pi '_f\right) \ \sim _{\mathbb {Q}(\Pi _f)\mathbb {Q}(\Pi '_f)} p(\Pi )\ p(\Pi ') \ p(m,\Pi _\infty ,\Pi '_\infty ) \ \mathcal {G}(\omega _{\Pi '_{f}}). \end{aligned}$$

In other words, the critical value $L(\tfrac{1}{2}+m,\Pi _f \times \Pi '_f)$ equals the product of three periods and the above Gauß-sum, up to multiplication by an element in the composition of rationality-fields $\mathbb {Q}(\Pi _f)\mathbb {Q}(\Pi '_f)$: These latter fields are defined by reference to the natural action of $\hbox {Aut}(\mathbb {C})$ on non-archimedean representations $\Pi _f$ and $\Pi '_f$ (see [32], §I.1), and, most importantly, they are number fields. Hence, our rationality-theorem [12, Thm. 3.9] amounts to a description of the transcendental part of $L(\tfrac{1}{2}+m,\Pi _f \times \Pi '_f)$, asserting that all critical values of $L(s,\Pi \times \Pi ')$ are a product of transcendental periods and a Gauß-sum, up to a factor coming out of a concrete number field, namely $\mathbb {Q}(\Pi _f)\mathbb {Q}(\Pi '_f)$, attached to $\Pi _f$ and $\Pi '_f$.

#### The main result of this paper

In this paper, we show that (1.1) is still true, if we enlarge our framework to

(i) general CM-fields *F*—instead of imaginary quadratic fields $\mathcal {K}$ and
(ii) general cohomological isobaric automorphic representations $\Pi '=\Pi _1\boxplus \cdots \boxplus \Pi _k$, which are fully-induced from distinct unitary cuspidal automorphic representation $\Pi _i$ of general linear groups $\mathrm{GL}_{n_i}(\mathbb {A}_F)$ of arbitrary rank $n_i\ge 1$—instead of sums of Hecke characters $\chi _i$.

In summary, our main result is

##### Theorem

Let *F* be any CM-field. Let $\Pi$ be a cuspidal automorphic representation of $\mathrm{GL}_n(\mathbb {A}_F)$, which is cohomological with respect to $\mathcal {E}_\mu$ and let $\Pi '=\Pi _1\boxplus \cdots \boxplus \Pi _k$ by an isobaric automorphic representation of $\mathrm{GL}_{n-1}(\mathbb {A}_F)$, fully induced from distinct unitary cuspidal automorphic representations $\Pi _i$, $1\le i\le k$, which is cohomological with respect to $\mathcal {E}_\lambda$ and of central character $\omega _{\Pi '}$. We assume that there is a non-trivial $\mathrm{GL}_{n-1}(F\otimes _\mathbb {Q}\mathbb {R})$-equivariant intertwining $\mathcal {E}_\mu \otimes \mathcal {E}_\lambda \rightarrow \mathbb {C}$. Then, for every critical point $\tfrac{1}{2}+m$ of $L(s,\Pi \times \Pi ')$, there is a non-zero archimedean period $p(m,\Pi _\infty ,\Pi '_\infty )\in \mathbb {C}^*$, only depending on *m*, $\Pi _\infty$ and $\Pi '_\infty$, such that

$$\begin{aligned} L\left( \tfrac{1}{2}+m,\Pi _f \times \Pi '_f\right) \ \sim _{\mathbb {Q}(\Pi _f)\mathbb {Q}(\Pi '_f)} p(\Pi )\ p(\Pi ') \ p(m,\Pi _\infty ,\Pi '_\infty ) \ \mathcal {G}(\omega _{\Pi '_{f}}), \end{aligned}$$

where “$\sim _{\mathbb {Q}(\Pi _f)\mathbb {Q}(\Pi '_f)}$” means up to multiplication by an element in the composition of number fields $\mathbb {Q}(\Pi _f)\mathbb {Q}(\Pi '_f)$.

Our main result has the following direct consequence:

##### Corollary

Let $\Pi$ and $\Pi '$ be as in the statement of the main theorem above. Let $\tfrac{1}{2}+m,\tfrac{1}{2}+\ell$ be two critical values of $L(s,\Pi \times \Pi ')$ and abbreviate $\Omega _{\Pi _\infty ,\Pi '_\infty }(m,\ell ):=p(m,\Pi _\infty ,\Pi '_\infty ) p(\ell ,\Pi _\infty ,\Pi '_\infty )^{-1}$. Then, whenever $L^S(\tfrac{1}{2}+\ell , \Pi \times \Pi ')$ is non-zero (e.g., if $\Pi$ is unitary and $\ell \ne 0$),

$$\begin{aligned} \frac{L^S\left( \tfrac{1}{2}+m, \Pi \times \Pi '\right) }{L^S\left( \tfrac{1}{2}+\ell , \Pi \times \Pi '\right) } \sim _{\mathbb {Q}(\Pi _f)\mathbb {Q}(\Pi '_f)} \Omega _{\Pi _\infty ,\Pi '_\infty }(m,\ell ), \end{aligned}$$

which only depends on the archimedean components $\Pi _\infty$ and $\Pi '_\infty$.

In particular, if $L^S(\tfrac{3}{2}+m, \Pi \times \Pi ')$ is non-zero (e.g., if $\Pi$ is unitary and $m\ne -1$), then the quotient of consecutive critical *L*-values satisfies

$$\begin{aligned} \frac{1}{\Omega _{\Pi _\infty ,\Pi '_\infty }(m)}\frac{L^S\left( \tfrac{1}{2}+m, \Pi \times \Pi '\right) }{L^S(\tfrac{3}{2}+m, \Pi \times \Pi ')} \in \mathbb {Q}(\Pi _f)\mathbb {Q}(\Pi '_f). \end{aligned}$$

Here we wrote $\Omega _{\Pi _\infty ,\Pi '_\infty }(m):=\Omega _{\Pi _\infty ,\Pi '_\infty }(m,m+1)$

As its key-feature, our corollary avoids any reference to Whittaker periods and expresses quotients of critical values of $L(s,\Pi \times \Pi ')$ in terms of archimedean factors only. The reader may want to compare this corollary to the main result of [15], where a similar result on quotients of consecutive critical values of Rankin–Selberg *L*-functions attached to cuspidal representations $\Pi$ and $\Pi '$ over totally real fields has been established. Our corollary hence complements this important result.

In order to keep our presentation precise, but at the same time short, we will focus on the crucial parts of the proof of our main theorem in this note and avoid repeating arguments given in [12] already, if they transfer verbatim to the more general situation here. In other words, we will only work out in details those steps of the proof, which need an extra argument, not contained in [12], and refer to precise statements in [12], if possible. *The reader is hence strongly advised to keep a copy of* [12] *ready at hand. Unexplained notation or references (e.g.,* “§*2.1.1*”) *refer to this source* [12].

### The setup

#### Algebraic data

We let *F* be any CM-field of dimension $2d = \dim _\mathbb {Q}F$ and set of archimedean places $S_\infty$. Each place $v\in S_\infty$ refers to a fixed pair of conjugate complex embeddings $(\iota _v,\bar{\iota }_v)$ of *F*, where we will drop the subscript “*v*” if it is clear from the context. We let $\mathcal {O}$ be the ring of integers of *F* and for $v\notin S_\infty$, $\mathcal {O}_v$ its local integral completion in $F_v$. The non-trivial additive character $\psi : F\backslash \mathbb {A}\rightarrow \mathbb {C}^*$ is defined as in §2.1.1. Throughout this note *G* denotes the general linear group $\mathrm{GL}_n$ and $G'$ denotes the general linear group $\mathrm{GL}_{n-1}$, both defined over *F* ($n\ge 2$).

#### Highest weight modules

We let $E_\mu$ (resp. $E_\lambda$) be an irreducible finite-dimensional representation of the *real* Lie group $G_\infty =R_{F/\mathbb {Q}}(G)(\mathbb {R})$ (resp. $G'_\infty =R_{F/\mathbb {Q}}(G')(\mathbb {R})$) on a complex vector-space, given by its highest weight $\mu =(\mu _v)_{v\in S_\infty }$ (resp. $\lambda =(\lambda _v)_{v\in S_\infty }$). Both representations are assumed to be algebraic: In terms of the standard choice of a maximal torus and positivity on the corresponding set of roots, this means that $\mu _v=(\mu _{\iota _v},\mu _{\bar{\iota }_v})\in \mathbb {Z}^n\times \mathbb {Z}^n$ (and the analogous assertion for $\lambda$). If $\sigma \in$ $\hbox {Aut}(\mathbb {C})$ is any automorphism of the field $\mathbb {C}$, then we define ${}^\sigma E_\mu$ to be the irreducible finite-dimensional representation of $G_\infty$ of highest weight ${}^\sigma \mu =(({}^\sigma \mu )_v)_{v\in S_\infty }$, where at a place $v=(\iota _v,\bar{\iota }_v)$ we let $({}^\sigma \mu )_v=(\mu _{\sigma ^{-1}\circ \iota _v},\mu _{\sigma ^{-1}\circ \bar{\iota }_v})$. The analogous definition yields us an irreducible finite-dimensional representation ${}^\sigma E_\lambda$ of $G'_\infty$.

#### Real unitary subgroups

We chose a maximal compact subgroup $C_\infty$ (resp. $C'_\infty$) of $G_\infty$ (resp. $G'_\infty$) and define real Lie subgroups $K_\infty :=Z_{G_\infty }C_\infty \cong (\mathbb {R}_{>0} U(n))^d$ of $G_\infty$ (resp. $K'_\infty :=Z_{G'_\infty }C'_\infty \cong (\mathbb {R}_{>0} U(n-1))^d$ of $G'_\infty$), where *U*(*k*) denotes the usual compact unitary Lie group of rank *k*.

#### The cuspidal representation $\Pi$

Throughout this note, $\Pi$ denotes a cuspidal automorphic representation of $G(\mathbb {A})$ with non-trivial $(\mathfrak {g}_\infty ,K_\infty )$-cohomology with respect to $E_\mu$: This is equivalent to $\Pi$ being regular algebraic in the sense of [6, Def. 3.12] (cf. [13, Thm. 6.3] for details). We do not assume $\Pi$ to be unitary, but allow arbitrary integer twists $\Vert \det \Vert ^\mathsf{m}$ of unitary cuspidal automorphic representations $\tilde{\Pi }$: $\Pi =\tilde{\Pi } \cdot \Vert \det \Vert ^\mathsf{m}$. For convenience we will not distinguish between a cuspidal automorphic representation, its smooth Fréchet space completion of moderate growth and its (non-smooth) Hilbert space completion in the $L^2$-spectrum. Introducing subindices “*v*”, $\Pi _\infty =\otimes _{v\in S_\infty }\Pi _v$ is hence locally of the form described in §2.4:

$$\begin{aligned} \Pi _v\cong \mathrm{Ind}_{B(\mathbb {C})}^{G(\mathbb {C})}\left[ z_1^{\ell _{v,1}+\mathsf{m}}\bar{z}^{-\ell _{v,1}+\mathsf{m}}_1\otimes \cdots \otimes z_n^{\ell _{v,n}+\mathsf{m}}\bar{z}^{-\ell _{v,n}+\mathsf{m}}_n\right] , \end{aligned}$$

where

$$\begin{aligned} \ell _{v,j}:=\ell (\mu _{\iota _v},j):=-\mu _{\iota _v,n-j+1}-\mathsf{m}+\frac{n+1}{2}-j \end{aligned}$$

and induction is unitary. By [6, Thm. 3.13], for each $\sigma \in \hbox {Aut}(\mathbb {C})$ there exists a unique cuspidal automorphic representation $^\sigma \Pi$ of $G(\mathbb {A})$, which is cohomological with respect to $^\sigma E_\mu$ and whose finite part satisfies $({}^\sigma \Pi )_f={}^\sigma (\Pi _f):=\Pi _f\otimes _{\sigma }\mathbb {C}$. Since $\mathsf{m}$ is an integer, we have ${}^\sigma \Pi =({}^\sigma \tilde{\Pi })\cdot \Vert \det \Vert ^\mathsf{m}$, where ${}^\sigma \tilde{\Pi }$ is a regular algebraic, unitary cuspidal automorphic representation, defined similarly. We let $W(\Pi _f)$ be the finite part of the global Whittaker model $W(\Pi ,\psi ^{-1})$ defined by the $\psi ^{-1}$-Fourier coefficient.

#### The isobaric representation $\Pi '$

Let $\sum _{i=1}^k n_i=n-1$ be any partition of $n-1$. As the second representation-theoretic ingredient, $\Pi '$ denotes an automorphic representation of $G'(\mathbb {A})$ with non-trivial $(\mathfrak {g}'_\infty ,K'_\infty )$-cohomology with respect to $E_\lambda$, which is the isobaric sum of pairwise different, unitary cuspidal automorphic representations $\Pi _i$ of $\mathrm{GL}_{n_i}(\mathbb {A})$, $1\le i\le k$,

$$\begin{aligned} \Pi ':=\Pi _1\boxplus \cdots \boxplus \Pi _k \cong \mathrm{Ind}_{P'(\mathbb {A})}^{G'(\mathbb {A})}[\Pi _1\otimes \cdots \otimes \Pi _k]. \end{aligned}$$

Here, $P'$ denotes the standard parabolic subgroup of $G'$ with Levi factor isomorphic to $\prod _{i=1}^k\mathrm{GL}_{n_i}$ (and the latter isomorphy of representations is automatic, [1, 2, 21, 31]).

##### Remark 1.1

As a paradigmatic example, any representation $\Pi '$ which is the cohomological quadratic base change from a quasi-split unitary group as in [7], p. 122, will be of the above form, see [7, Thm. 6.1].

Since the cuspidal representations $\Pi _i$ are pairwise different, a combination of [29, Prop. 7.1.3, Thm. 3.5.12 and Rem. 3.5.14] implies that $\Pi '$ is globally $\psi$-generic. We let $W(\Pi '_f)$ be the finite part of the global Whittaker model $W(\Pi ',\psi )$ defined by the $\psi$-Fourier coefficient.

Abstract local genericity of the irreducible unitary representations $\Pi '_v$ at an archimedean place $v\in S_\infty$ hence shows (cf., e.g., [13] §5.5) that necessarily

$$\begin{aligned} \Pi '_v\cong \mathrm{Ind}_{B'(\mathbb {C})}^{G'(\mathbb {C})}\left[ z_1^{k_{v,1}}\bar{z}^{-k_{v,1}}_1\otimes \cdots \otimes z_{n-1}^{k_{v,n-1}}\bar{z}^{-k_{v,n-1}}_{n-1}\right] , \end{aligned}$$

where

$$\begin{aligned} k_{v,j}:=k(\lambda _{\iota _v},j):=-\lambda _{\iota _v,n-j}+\frac{n}{2}-j, \end{aligned}$$

i.e., each $\Pi '_v$ is of the form considered in §2.5.

Let $\rho _{P'}$ be the usual square-root of the modulus character of $P'(\mathbb {A})$, [5, 0, 3.5]. We write $\rho _i:=\rho _{P'}|_{\mathrm{GL}_{n_i}}$ for the restriction of $\rho _{P'}$ to the particular factor $\mathrm{GL}_{n_i}$ of the Levi subgroup. By [5, III, Thm. 3.3] the global representations $\Xi _i:=\Pi _i\cdot \rho _i$ are regular algebraic cuspidal automorphic representations (for details see [13, pp. 1002–1003]). Hence, as for $\Pi$ above, for each $\sigma \in$ $\hbox {Aut}(\mathbb {C})$ and all $1\le i\le k$, there are uniquely determined cuspidal automorphic representations ${}^\sigma \Xi _i$, which are cohomological with respect to the corresponding, $\sigma$-permuted coefficient module of $\mathrm{GL}_{n_i}(\mathbb {C})$ and whose finite part satisfies $({}^\sigma \Xi _i)_f={}^\sigma (\Xi _{i,f}):=\Xi _{i,f}\otimes _{\sigma }\mathbb {C}$. The representations $({}^\sigma \Xi _i)\cdot \rho ^{-1}_i$ are hence pairwise different, unitary cuspidal automorphic representations. We let

$$\begin{aligned} {}^\sigma \Pi ':=({}^\sigma \Xi _1)\cdot \rho ^{-1}_1\boxplus \cdots \boxplus ({}^\sigma \Xi _k)\cdot \rho ^{-1}_k \end{aligned}$$

be their isobaric sum.

##### Lemma 1.2

The representation ${}^\sigma \Pi '$ is fully induced, i.e.,

$$\begin{aligned} {}^\sigma \Pi '=({}^\sigma \Xi _1)\cdot \rho ^{-1}_1\boxplus \cdots \boxplus ({}^\sigma \Xi _k)\cdot \rho ^{-1}_k\cong \mathrm{Ind}_{P'(\mathbb {A})}^{G'(\mathbb {A})}\left[ ({}^\sigma \Xi _{1})\cdot \rho ^{-1}_{1}\otimes \cdots \otimes ({}^\sigma \Xi _{k})\cdot \rho ^{-1}_{k}\right] \end{aligned}$$

and we have $({}^\sigma \Pi ')_f\cong {}^\sigma (\Pi '_{f})$.

##### Proof

For the first assertion observe that $({}^\sigma \Xi _{i,v})\cdot \rho ^{-1}_{i,v}$ is irreducible and unitary for each $1\le i\le k$ and each place *v* of *F*. Hence, $\mathrm{Ind}_{P'(F_v)}^{G'(F_v)}[({}^\sigma \Xi _{1,v})\cdot \rho ^{-1}_{1,v}\otimes \cdots \otimes ({}^\sigma \Xi _{k,v})\cdot \rho ^{-1}_{k,v}]$ is irreducible for each *v* of *F*, see [2] (for $v\notin S_\infty$) and [1, 31] (for $v\in S_\infty$). It follows that

$$\begin{aligned}&\mathrm{Ind}_{P'(\mathbb {A})}^{G'(\mathbb {A})}[({}^\sigma \Xi _{1})\cdot \rho ^{-1}_{1}\otimes \cdots \otimes ({}^\sigma \Xi _{k})\cdot \rho ^{-1}_{k}]\cong \otimes _v \mathrm{Ind}_{P'(F_v)}^{G'(F_v)}[({}^\sigma \Xi _{1,v})\\&\quad \cdot \rho ^{-1}_{1,v}\otimes \cdots \otimes ({}^\sigma \Xi _{k,v})\cdot \rho ^{-1}_{k,v}] \end{aligned}$$

is irreducible as well. Hence, ${}^\sigma \Pi '=({}^\sigma \Xi _1)\cdot \rho ^{-1}_1\boxplus \cdots \boxplus ({}^\sigma \Xi _k)\cdot \rho ^{-1}_k$ being isomorphic to a non-trivial subquotient of the latter global, induced representation, cf. [21], p. 208, shows that

$$\begin{aligned} {}^\sigma \Pi '=({}^\sigma \Xi _1)\cdot \rho ^{-1}_1\boxplus \cdots \boxplus ({}^\sigma \Xi _k)\cdot \rho ^{-1}_k\cong \mathrm{Ind}_{P'(\mathbb {A})}^{G'(\mathbb {A})}\left[ ({}^\sigma \Xi _{1})\cdot \rho ^{-1}_{1}\otimes \cdots \otimes ({}^\sigma \Xi _{k})\cdot \rho ^{-1}_{k}\right] . \end{aligned}$$

For the second claim, observe that at $v\notin S_\infty$, the action of $\sigma \in$ $\hbox {Aut}(\mathbb {C})$ commutes with unnormalized, algebraic induction “${}^a\mathrm{Ind}$”, i.e., one has

$$\begin{aligned} {}^\sigma \mathrm{Ind}_{P'(F_v)}^{G'(F_v)}[\Pi _{1,v}\otimes \cdots \otimes \Pi _{k,v}]= & {} \mathrm{Ind}_{P'(F_v)}^{G'(F_v)}[\Pi _{1,v}\otimes \cdots \otimes \Pi _{k,v}]\otimes _\sigma \mathbb {C}\\\cong & {} {}^{a}\mathrm{Ind}_{P'(F_v)}^{G'(F_v)}[(\Xi _{1,v}\otimes _\sigma \mathbb {C})\otimes \cdots \otimes (\Xi _{k,v}\otimes _\sigma \mathbb {C})]\\\cong & {} \mathrm{Ind}_{P'(F_v)}^{G'(F_v)}[({}^\sigma \Xi _{1,v})\cdot \rho ^{-1}_{1,v}\otimes \cdots \otimes ({}^\sigma \Xi _{k,v})\cdot \rho ^{-1}_{k,v}], \end{aligned}$$

This completes the proof. $\square$

As a consequence of Lemma 1.2, reading [5, III, Thm. 3.3] backwards shows that ${}^\sigma \Pi '$ is cohomological with respect to ${}^\sigma E_\lambda$. Moreover, the same argument as above shows that ${}^\sigma \Pi '$ is globally $\psi$-generic for all $\sigma \in \hbox {Aut}(\mathbb {C})$.

Hence, ${}^\sigma \Pi '$ satisfies the same properties imposed on $\Pi '$ above, i.e., $\hbox {Aut}(\mathbb {C})$ leaves the class of $(\mathfrak {g}'_\infty ,K'_\infty )$-cohomological isobaric sums of pairwise different, unitary cuspidal automorphic representations stable.

### Differences to the imaginary quadratic case: archimedean considerations

#### Highest weight representations carrying cuspidal data

Let $E_\mu$ be a coefficient module as in Sect. 1.2.4, i.e., $H^*(\mathfrak {g}_\infty ,K_\infty \Pi \otimes E_\mu )\ne 0$ for a cuspidal representation $\Pi$ as described above. This implies strong restrictions on the highest weight $\mu =(\mu _v)_{v\in S_\infty }$ in terms of its local components at archimedean places (which we may now have in an arbitrary number $d=|S_\infty |$), which we summarize shortly as

##### Lemma 1.3

1. $$\mu _{\iota _v}-\mu ^\mathsf{v}_{\bar{\iota }_v}=(-2 \mathsf{m},\ldots ,-2\mathsf{m})$$ for all $$v\in S_\infty $$.
2. $({}^\sigma \mu )_{\bar{\iota }_v}=\mu _{\overline{\sigma ^{-1}\circ \iota _v}}$ for all $v\in S_\infty$ and all $\sigma \in \mathrm{Aut}(\mathbb {C})$.

##### Proof

1. By assumption $E_\mu$ supports non-zero cohomology with respect to the cuspidal representation $\Pi =\tilde{\Pi }\cdot \Vert \det \Vert ^\mathsf{m}$, where $\tilde{\Pi }$ is unitary. Hence, $E_{\mu +\mathsf{m}}$ is conjugate self-dual by [5, I, Cor. 4.2] and [4, Lem. 1.3]. This implies (1).
2. Let $\sigma \in \mathrm{Aut}(\mathbb {C})$. The irreducible module ${}^\sigma E_\mu$ of highest weight $^\sigma \mu =(({}^\sigma \mu )_v)_{v\in S_\infty }$ supports non-zero $(\mathfrak {g}_\infty ,K_\infty )$-cohomology with respect to the cuspidal automorphic representation ${}^\sigma \Pi$. Since ${}^\sigma \Pi =({}^\sigma \tilde{\Pi })\cdot \Vert \det \Vert ^\mathsf{m}$, our point (1) above implies that $({}^\sigma \mu )_{\iota _v}-({}^\sigma \mu )^\mathsf{v}_{\bar{\iota }_v}=(-2 \mathsf{m},\ldots ,-2\mathsf{m})$ for all $v\in S_\infty$ and the same integer $\mathsf{m}$ for all $\sigma$. Inserting the definition of ${}^\sigma \mu$ gives
  $$\begin{aligned} \mu _{\sigma ^{-1}\circ \iota _v,j}+\mu _{\sigma ^{-1}\circ \bar{\iota }_v,n-j+1}=-2\mathsf{m}. \end{aligned}$$
  for all $1\le j \le n$. On the other hand, applying (1) to the embedding $\iota '_v:=\sigma ^{-1}\circ \iota _v$ of *F*, we obtain
  $$\begin{aligned} \mu _{\sigma ^{-1}\circ \iota _v,j}+\mu _{\overline{\sigma ^{-1}\circ \iota _v},n-j+1}=-2\mathsf{m} \end{aligned}$$
  for all $1\le j \le n$. Combining the latter two equations shows $\mu _{\sigma ^{-1}\circ \bar{\iota }_v,n-j+1}=\mu _{\overline{\sigma ^{-1}\circ \iota _v},n-j+1}$ for every *j* and arbitrary $v\in S_\infty$, and $\sigma \in$ $\hbox {Aut}(\mathbb {C})$. This proves (2).

$$\square $$

#### Cohomological automorphic representations

Although maybe looking as a pure technicality at first, Lemma 1.3 (2) is an important assertion: It guarantees that the action of $\hbox {Aut}(\mathbb {C})$ on those coefficient modules $E_\mu$ and $E_\lambda$, which carry automorphic cohomology as in Sects. 1.2.4 and 1.2.5,—although defined abstractly as a potentially arbitrary permutation of all the embeddings $\iota : F\hookrightarrow \mathbb {C}$—does not tear apart the data $(\mu _{\iota _v},\mu _{\bar{\iota }_v})$ resp. $(\lambda _{\iota _v},\lambda _{\bar{\iota }_v})$ which is attached to a pair of embeddings $(\iota _v,\bar{\iota }_v)$ forming an archimedean place *v*. This implies the following corollary, which says that $\hbox {Aut}(\mathbb {C})$ acts on $\Pi _\infty$ and $\Pi '_\infty$ simply as a permutation of the local factors, potentially followed by a conjugation of the characters forming the inducing data:

##### Corollary 1.4

For $\sigma \in \mathrm{Aut}(\mathbb {C})$, let ${}^\sigma \ell _{v}:=\ell (({}^\sigma \mu )_{\iota _v},j)=\ell (\mu _{\sigma ^{-1}\circ \iota _v},j)$ and ${}^\sigma k_{v}:=k(({}^\sigma \lambda )_{\iota _v},j)=k(\lambda _{\sigma ^{-1}\circ \iota _v},j)$. For the archimedean components of the automorphic representations $^\sigma \Pi$ and $^\sigma \Pi '$, we obtain

1. $$(^\sigma \Pi )_\infty \cong \otimes _{v\in S_\infty } \mathrm{Ind}_{B(\mathbb {C})}^{G(\mathbb {C})}\left[ z_1^{{}^\sigma \ell _{v,1}+\mathsf{m}}\bar{z}^{-{}^\sigma \ell _{v,1}+\mathsf{m}}_1\otimes \cdots \otimes z_n^{{}^\sigma \ell _{v,n}+\mathsf{m}}\bar{z}^{-{}^\sigma \ell _{v,n}+\mathsf{m}}_n\right] $$
2. $$(^\sigma \Pi ')_\infty \cong \otimes _{v\in S_\infty } \mathrm{Ind}_{B'(\mathbb {C})}^{G'(\mathbb {C})}\left[ z_1^{{}^\sigma k_{v,1}}\bar{z}^{-{}^\sigma k_{v,1}}_1\otimes \cdots \otimes z_{n-1}^{{}^\sigma k_{v,n-1}}\bar{z}^{-{}^\sigma k_{v,n-1}}_{n-1}\right] $$

##### Proof

For $\Pi$ this follows from Lemma 1.3, [5, IV Lem. 4.9] and the uniqueness of irreducible unitary generic representations of $\mathrm{GL}_r(\mathbb {C})$, $r\ge 1$, with non-trivial cohomology with respect to a given finite-dimensional coefficient module, cf. [8, Thm. 6.1] (See also [13, §5.5] for a detailed exposition of the latter assertion). For $\Pi '$ one first applies what we just said about $\Pi$ to the cuspidal datum $\Xi _1$, ..., $\Xi _k$ and then carefully uses [5, III, Thm. 3.3] together with induction in stages. $\square$

As a final consequence, and this is establishes the purpose of this section, we derive the following

**“Meta-Lemma”** Let $\hbox {A}_\infty$ be an assertion of first-order predicate calculus, involving only $^\sigma \Pi _\infty$ or $^\sigma \Pi '_\infty$ for a family of $\sigma \in$ $\hbox {Aut}(\mathbb {C})$. If $\hbox {A}_\infty$ is true if and only if its restriction $\hbox {A}_v$ to $^\sigma \Pi _v$ and $^\sigma \Pi '_v$ is true for all $v\in S_\infty$, and $\hbox {A}_v$ is shown by an argument in [12], then $\hbox {A}_\infty$ holds.

#### Archimedean consequences of the Meta-Lemma

Making our choices place-by-place $v\in S_\infty$ and applying our meta-lemma, we obtain

1. A natural $\mathbb {Q}(E_\mu )$-rational vector-space structure on $H^q(\mathfrak {g}_\infty ,K_\infty ,\Pi _\infty \otimes E_\mu )$ (resp. $\mathbb {Q}(E_\lambda )$-rational vector-space structure on $H^q(\mathfrak {g}'_\infty ,K'_\infty ,\Pi '_\infty \otimes E_\lambda )$) as in §2.7.
2. Basis-vectors $[\Pi _\infty ]$ (resp. $[\Pi '_\infty ]$) of the one-dimensional spaces $H^{b_n}(\mathfrak {g}_\infty ,K_\infty ,W(\Pi _\infty )\otimes E_\mu )$ (resp. $H^{b_{n-1}}(\mathfrak {g}'_\infty ,K'_\infty ,W(\Pi '_\infty )\otimes E_\lambda )$), where $b_r=d\cdot \frac{r(r-1)}{2}$, as in §3.3.
3. A well-defined “interlacing-hypothesis” of the highest weights $\mu$ and $\lambda$ as in Hypothesis 2.3: This means we assume the validity of

##### Hypothesis 1.5

For all archimedean places $v=(\iota _v,\bar{\iota }_v)$ the following inequalities hold:

$$\begin{aligned} \mu _{\iota _v,1}\ge & {} -\lambda _{\iota _v,n-1}\ge \mu _{\iota _v,2}\ge -\lambda _{\iota _v,n-2}\ge \cdots \ge -\lambda _{\iota _v,1}\ge \mu _{\iota _v,n}\\ \mu ^\mathsf{v}_{\bar{\iota }_v,1}\ge & {} -\lambda ^\mathsf{v}_{\bar{\iota }_v,n-1}\ge \mu ^\mathsf{v}_{\bar{\iota }_v,2}\ge -\lambda ^\mathsf{v}_{\bar{\iota }_v,n-2}\ge \cdots \ge -\lambda ^\mathsf{v}_{\bar{\iota }_v,1}\ge \mu ^\mathsf{v}_{\bar{\iota }_v,n}. \end{aligned}$$

(4) Given (the well-definedness of) this hypothesis, a description of the set of critical points Crit$(\Pi \times \Pi ')\subset \tfrac{1}{2}+\mathbb {Z}$ of $L(s,\Pi \times \Pi ')$:
  $$\begin{aligned} \tfrac{1}{2}+m\in \mathrm{Crit}(\Pi \times \Pi ') \quad \Leftrightarrow \quad \mathrm{Hom}_{R_{F/\mathbb {Q}}(G')(\mathbb {C})}(E_{\mu -m}\otimes E_\lambda ,\mathbb {C})\ne 0. \end{aligned}$$
  The proof proceeds as in Lem. 3.5, though, one needs to correct a slight mistake *ibidem* first: The restriction to non-negative $m\ge 0$ there is not to be made. See also Thm. 2.21 in [25], where this has meanwhile been proved in even greater generality.
(5) For all $\tfrac{1}{2}+m\in$Crit$(\Pi \times \Pi ')$, compatible choices of intertwining operators
  $\mathcal {T}^{(m)}\in \mathrm{Hom}_{R_{F/\mathbb {Q}}(G')(\mathbb {C})}(E_{\mu -m}\otimes E_\lambda ,\mathbb {C})$ as in §3.7. Again, following the previous point, there is no restriction on *m* being positive or negative here.
(6) Finally and most importantly, for all $\tfrac{1}{2}+m\in$Crit$(\Pi \times \Pi ')$, well-defined complex numbers $c(\tfrac{1}{2}+m,\Pi _\infty ,\Pi '_\infty )$, defined as in §3.10, and proved to be non-vanishing as in Thm. 3.8. This allows us to define archimedean periods $p(m,{}^\sigma \Pi _\infty ,{}^\sigma \Pi '_\infty )$ as in §3.10, i.e., as the inverse of $c(\tfrac{1}{2}+m,{}^\sigma \Pi _\infty ,{}^\sigma \Pi '_\infty )$, for all $\sigma \in$ $\hbox {Aut}(\mathbb {C})$. As it has been discussed above, this works whether or not $m\ge 0$.

### Differences to the imaginary quadratic case: non-archimedean considerations

#### Special Whittaker vectors

We will choose very particular vectors $\xi _{\Pi '_v}\in W(\Pi '_v)$, at all non-archimedean places $v\notin S_\infty$ in analogy to §3.9. Let $T'\subset B'\subset G'$ be the diagonal maximal torus in the standard Borel subgroup $B'$ of $G'$ and denote $T'(F_v)^+:=\{t\in T'(F_v)| t_i t_{i+1}^{-1}\in \mathcal {O}_v, t_{n-1}=1\}$. Since $\Pi '_v$ is the generic, the assumptions of [20, Proposition (3.2)] are satisfied. Hence, any non-vanishing functional $\xi _{\Pi '_v}\in W(\Pi '_v)$ is already non-zero on $T'(F_v)^+\subset G'(F_v)$. As another ingredient, let $K'(m'_v)$ be the mirahoric subgroup of $G'(F_v)$ of level $m'_v$. If $m'_v$ equals the conductor of $\Pi '_v$, then, by [17, Theorem (5.1)] the space of Whittaker vectors, transforming by the central character $\omega _{\Pi '_v}$ of $\Pi '_v$ under the $K'(m'_v)$ is one-dimensional, its elements being called *new vectors*. As a consequence of the above discussion, we may fix a matrix $t_{\Pi '_v}\in T'(F_v)^+$ on which all the non-trivial new vectors of $\Pi '_v$ do not vanish simultaneously, where we observe that we may choose the same matrix for all $\sigma$-twists of $\Pi '_v$, i.e., such that $t_{\Pi '_v}=t_{{}^\sigma \Pi '_v}$. Moreover, if the non-archimedean place *v* is outside the set of ramification of $\Pi '$ and $\psi$, then we may take $t_{\Pi '_v}:=id$. Depending on these (mild) choices, for all $v\notin S_\infty$, we define $\xi _{\Pi '_v}\in W(\Pi '_v)$ to be the unique new vector such that $\xi _{\Pi '_v}(t_{\Pi '_v})=1$.

As the last ingredient, we remark that we may similarly also choose particular Whittaker vectors $\xi _{\Pi _v}$ for $\Pi _v$, $v\notin S_\infty$: These choices depend on our data fixed for $\Pi '_v$ above and can be made, *mutatis mutandis*, precisely as in §3.9: First, we fix a matrix $t_{\Pi _v}\in T(F_v)^+$, analogously as for $G'(F_v)$. Now, for a non-archimedean place *v* outside the set of ramification of $\Pi '$ and $\psi$, we let $\xi _{\Pi _v}$ be the unique new vector of $\Pi _v$, which satisfies $\xi _{\Pi _v}(t_{\Pi _v})=1$. It is a certain, non-zero multiple $c_{\Pi _v}$ of the essential vector, see [17, (4.1) Théorème]. If *v* is, however, inside the set of ramification of $\Pi '$ or $\psi$, then we take $\xi _{\Pi _v}$ to be the unique Whittaker vector, whose restriction to $G'(F_v)$ is supported on $N'(F_v)t_{\Pi '_v} K'(m'_v)$ and there equal to $\psi ^{-1}_v\omega ^{-1}_{\Pi '_v}$. See also [26, 3.1.4] and [22, 2.1.1], where such choices were coined first.

Finally, we observe that Lemma 3.7 still holds for these special Whittaker vectors.

#### Rational structures for Whittaker models

Keeping in mind the above considerations, we see as in Prop. 2.7 that the representations $W(\Pi _f)$ and $W(\Pi '_f)$ may be defined over the rationality fields $\mathbb {Q}(\Pi _f)$, respectively $\mathbb {Q}(\Pi '_f)$, by taking invariants of normalized new vectors in each model. Moreover, both fields $\mathbb {Q}(\Pi _f)$ and $\mathbb {Q}(\Pi '_f)$ are number fields by the regular-algebraicity of the cuspidal representations $\Pi$ and $\Xi _1$, ...$\Xi _k$, see [6, Thm. 3.13] (or, for a detailed proof, [13, Thm. 8.1]).

### Global considerations

#### Eisenstein cohomology

We let $S_n:= G(F)\backslash G(\mathbb {A})/K_\infty$, $S_{n-1}:=G'(F)\backslash G'(\mathbb {A})/K'_\infty$ and $\tilde{S}_{n-1}:= G'(F)\backslash G'(\mathbb {A})/C'_\infty \cong S_{n-1}\times \mathbb {R}^d_+$, similar to §3.1. These spaces are orbifolds and we have $\dim _\mathbb {R}(\tilde{S}_{n-1})=b_n+b_{n-1}$.

We define $\varphi _{P'}$ to be the associate class of cuspidal automorphic representations of $L'(\mathbb {A})$, which is defined by the unitary cuspidal $\tau :=\Pi _1\otimes \cdots \otimes \Pi _k$. The space $\mathcal {A}_{\mathcal {J}',\{P'\},\varphi _{P'}}$ of automorphic forms is then defined as in §3.1. See also the original source [10, §1.3] or [11, §2.3]. We obtain the following important result on Eisenstein cohomology:

##### Proposition 1.6

The natural morphism

$$\begin{aligned} \imath ^{b_{n-1}}_{\Pi '}: H^{b_{n-1}}(\mathfrak {g}'_\infty ,K'_\infty , \Pi '\otimes E_\lambda )\rightarrow H^{b_{n-1}}(\mathfrak {g}'_\infty ,K'_\infty , \mathcal {A}_{\mathcal {J}',\{P'\},\varphi _{P'}}\otimes E_\lambda ) \end{aligned}$$

of $G(\mathbb {A}_f)$-modules, induced by the natural injection $\imath _{\Pi '}:\Pi '\hookrightarrow \mathcal {A}_{\mathcal {J}',\{P'\},\varphi _{P'}}$, is an isomorphism. Hence, there is the following commuting triangle of natural injections of $G'(\mathbb {A}_f)$-modules


##### Proof

We assume familiarity with the general results of [11]. In [11, §3.1], following [9], a filtration

1.2 $$\begin{aligned} \mathcal {A}_{\mathcal {J}',\{P'\},\varphi _{P'}}=\mathcal {A}^{(0)}_{\mathcal {J}',\{P'\},\varphi _{P'}}\supseteq \mathcal {A}^{(1)}_{\mathcal {J}',\{P'\},\varphi _{P'}}\supseteq \cdots \supseteq \mathcal {A}^{(m)}_{\mathcal {J}',\{P'\},\varphi _{P'}} \end{aligned}$$

of $\mathcal {A}_{\mathcal {J}',\{P'\},\varphi _{P'}}$ of finite length $m=m(\{P'\})$ has been defined. The successive quotients are shown to be isomorphic to a direct sum, index by a set of (isomorphism classes) of quadruples in $M^{(j)}_{\mathcal {J}',\{P'\},\varphi _{P'}}$, $0\le j \le m$. See [11, Thm. 4] for this result and [11, §3.2] for a precise definition of $M^{(j)}_{\mathcal {J}',\{P'\},\varphi _{P'}}$. By construction (of the filtration (1.2) and of the sets $M^{(j)}_{\mathcal {J}',\{P'\},\varphi _{P'}}$), one necessarily finds $(P',\tau ,0,0)\in M^{(m)}_{\mathcal {J}',\{P'\},\varphi _{P'}}$, cf. [11, §3.1–3.2]. However, as all summands in $\Pi '$ are different and unitary, the description of the residual spectrum of $\mathrm{GL}_N$, cf. [24] II–III, implies that this is the only quadruple at all, i.e., $\cup _{j=0}^m M^{(j)}_{\mathcal {J}',\{P'\},\varphi _{P'}} = \{(P',\tau ,0,0)\}$. As a consequence, see again [11, Thm. 4] in combination with Mulitplicity One for the discrete spectrum of $G'(\mathbb {A})$,

$$\begin{aligned} \mathcal {A}_{\mathcal {J}',\{P'\},\varphi _{P'}}= & {} \mathcal {A}^{(0)}_{\mathcal {J}',\{P'\},\varphi _{P'}}= \mathcal {A}^{(1)}_{\mathcal {J}',\{P'\},\varphi _{P'}}=\cdots =\mathcal {A}^{(m)}_{\mathcal {J}',\{P'\},\varphi _{P'}}\\\cong & {} \mathrm{Ind}_{P'(\mathbb {A})}^{G'(\mathbb {A})}\left[ \tau \otimes S(\check{\mathfrak {a}}_{P',\mathbb {C}}^{G'})\right] , \end{aligned}$$

where $S(\check{\mathfrak {a}}_{P',\mathbb {C}}^{G'})$ is the symmetric algebra of the dual of the Lie algebra of the split component $A_{P'}$ of $P'$, modulo the split component of $G'$. Hence,

$$\begin{aligned} H^{q}\left( \mathfrak {g}'_\infty ,K'_\infty , \mathcal {A}_{\mathcal {J}',\{P'\},\varphi _{P'}}\otimes E_\lambda \right) \cong H^{q}\left( \mathfrak {g}'_\infty ,K'_\infty , \mathrm{Ind}_{P'(\mathbb {A})}^{G'(\mathbb {A})}\left[ \tau \otimes S(\check{\mathfrak {a}}_{P',\mathbb {C}}^{G'})\right] \otimes E_\lambda \right) , \end{aligned}$$

for all degrees *q*, see also [11, Cor. 16]. By the minimality of the degree $q=b_{n-1}$, we obtain

$$\begin{aligned} H^{b_{n-1}}(\mathfrak {g}'_\infty ,K'_\infty , \mathcal {A}_{\mathcal {J}',\{P'\},\varphi _{P'}}\otimes E_\lambda )\cong \Pi '_f, \end{aligned}$$

see [13] (7.25), revealing $H^{b_{n-1}}(\mathfrak {g}'_\infty ,K'_\infty , \mathcal {A}_{\mathcal {J}',\{P'\},\varphi _{P'}}\otimes E_\lambda )$ as irreducible. The natural map in cohomology $\imath ^{b_{n-1}}_{\Pi '}$ induced from the natural inclusion $\imath _{\Pi '}: \Pi '\hookrightarrow \mathcal {A}_{\mathcal {J}',\{P'\},\varphi _{P'}}$ has by construction the same image as the map in cohomology induced from the Eisenstein summation map

$$\begin{aligned} \mathrm{Eis}_0: \mathrm{Ind}_{P'(\mathbb {A})}^{G'(\mathbb {A})}[\tau ] {\mathop {\longrightarrow }\limits ^{\sim }}\Pi ', \end{aligned}$$

cf. [21]. Hence, recalling that all Eisenstein series attached to $K_\infty$-finite sections in $\mathrm{Ind}_{P'(\mathbb {A})}^{G'(\mathbb {A})}[\tau ]$ are holomorphic at $\Lambda =0$, $\imath ^{b_{n-1}}_{\Pi '}$ is non-zero by [28], Satz 4.11. See also [3] 2.9. As $H^{b_{n-1}}(\mathfrak {g}'_\infty ,K'_\infty , \mathrm{Ind}_{P'(\mathbb {A})}^{G'(\mathbb {A})}[\tau ]\otimes E_\lambda )\cong \Pi '_f$ is irreducible, too, by the minimality of $q=b_{n-1}$, $\imath ^{b_{n-1}}_{\Pi '}$ is an isomorphism. Now define $\mathscr {F}^{b_{n-1}}_\lambda$ to be the restriction to $H^{b_{n-1}}(\mathfrak {g}'_\infty ,K'_\infty , \mathcal {A}_{\mathcal {J}',\{P'\},\varphi _{P'}}\otimes E_\lambda )$ of the isomorphism of [9, Thm. 18] and $\Psi _{\Pi '}^\mathsf{Eis}:=\mathscr {F}^{b_{n-1}}_\lambda \circ \imath ^{b_{n-1}}_{\Pi '}$. Recalling the direct sum decomposition of Eisenstein cohomology, cf. [10, Thm. 2.3] or [11, §4.1–4.3] shows that $\mathscr {F}^{b_{n-1}}_\lambda$ (and hence also $\Psi _{\Pi '}^\mathsf{Eis}$) are injections. $\square$

#### Rational structures on submodules of automorphic cohomology and related Whittaker periods

As a consequence of the previous section, the following global results and assertions transfer from [12]: firstly, we obtain

##### Proposition 1.7

For any $\sigma \in \mathrm{Aut}(\mathbb {C})$ the natural $\sigma$-linear bijection $\tilde{\sigma }:\!\! H^{b_{n-1}}(S_{n-1},\mathcal {E}_\lambda )\rightarrow H^{b_{n-1}}(S_{n-1},^\sigma \mathcal {E}_\lambda )$ maps the image of $\Psi _{\Pi '}^\mathsf{Eis}$ onto the image of $\Psi _{^\sigma \Pi '}^\mathsf{Eis}$.

##### Proof

Let ${}^\sigma \varphi _{P'}$ be the associate class of the unitary cuspidal automorphic reprepsentation $^\sigma \tau :=({}^\sigma \Xi _1)\cdot \rho ^{-1}_{i}\otimes \cdots \otimes ({}^\sigma \Xi _k)\cdot \rho ^{-1}_{k}$. By its very definition $^\sigma \Pi '$ is the isobaric automorphic sum of the unitary cuspidal automorphic representations $({}^\sigma \Xi _i)\cdot \rho ^{-1}_{P'}$, from which it is fully-induced, see Lemma 1.2. Applying Proposition 1.6 to $\Pi '$ and $^\sigma \Pi '$ reduces the problem to showing that $\tilde{\sigma }: H^{b_{n-1}}(S_{n-1},\mathcal {E}_\lambda )\rightarrow H^{b_{n-1}}(S_{n-1},^\sigma \mathcal {E}_\lambda )$ maps $\mathscr {F}^{b_{n-1}}_\lambda (H^{b_{n-1}}(\mathfrak {g}'_\infty ,K'_\infty , \mathcal {A}_{\mathcal {J}',\{P'\},\varphi _{P'}}\otimes E_\lambda ))$ onto the analogously defined module $\mathscr {F}^{b_{n-1}}_{{}^\sigma \lambda }(H^{b_{n-1}}(\mathfrak {g}'_\infty ,K'_\infty , \mathcal {A}_{\mathcal {J}',\{P'\},{}^\sigma \varphi _{P'}}\otimes {}^\sigma E_\lambda ))$. However, using that $\imath ^{b_{n-1}}_{\Pi '}$ and $\imath ^{b_{n-1}}_{{}^\sigma \Pi '}$ are isomorphisms, i.e., invoking Proposition 1.6 once more, exactly the same arguments as in [13, proof of Thm. 7.23] go through, where this assertion is proved for regular coefficients $E_\lambda$. This shows the claim. $\square$

##### Definition 1.8

As a consequence of Propositions 1.6 and 1.7 the composition $(\Psi _{{}^\sigma \Pi '}^\mathsf{Eis})^{-1}\circ \tilde{\sigma }\circ \Psi _{\Pi '}^\mathsf{Eis}$ makes sense and we denote the resulting $\sigma$-linear bijection

$$\begin{aligned} H^{b_{n-1}}(\mathfrak {g}'_\infty ,K'_\infty , \Pi '\otimes E_\lambda )\rightarrow H^{b_{n-1}}(\mathfrak {g}'_\infty ,K'_\infty , ^\sigma \Pi '\otimes {}^\sigma E_\lambda ) \end{aligned}$$

again by $$\tilde{\sigma }$$.

As an immediate corollary, we obtain a $\mathbb {Q}(\Pi '_f)$-structure on the image of the injection $\Psi _{\Pi '}^\mathsf{Eis}$, which naturally extends the $\mathbb {Q}(E_\lambda )$-structure of $H^{b_{n-1}}(S_{n-1},\mathcal {E}_\lambda )$ defined by Betti-cohomology: This follows easily from Propositions 1.7 above, invoking [6, Lem. 3.2.1] (and recalling that $\mathbb {Q}(E_\lambda )\subseteq \mathbb {Q}(\Pi '_f)$, which ones concludes exactly as in the proof of [13, Cor. 8.7]). Hence, by transfer of structure along the injection $\Psi _{\Pi '}^\mathsf{Eis}$, constructed in Proposition 1.6, the irreducible $G'(\mathbb {A}_f)$-module $H^{b_{n-1}}(\mathfrak {g}'_\infty ,K'_\infty , \Pi '\otimes E_\lambda )$ carries a $\mathbb {Q}(\Pi '_f)$-structure. We assume from now on to have fixed precisely this rational structure on the cohomology of $\Pi '$ (and analogously on all its $\sigma$-twists $^\sigma \Pi '$).

Similarly, as it is well-known, the same arguments apply for the cuspidal automorphic representation $\Pi$ and its $(\mathfrak {g}_\infty ,K_\infty )$-cohomology, which injects into $H^{b_{n}}(S_{n},\mathcal {E}_\mu )$: We obtain a $\mathbb {Q}(\Pi _f)$-structure on $H^{b_n}(\mathfrak {g}_\infty ,K_\infty ,\Pi \otimes E_\mu )$, which naturally extends the $\mathbb {Q}(E_\mu )$-structure of $H^{b_{n}}(S_{n},\mathcal {E}_\mu )$ defined by Betti-cohomology and a natural $\sigma$-linear bijection $\tilde{\sigma }: H^{b_n}(\mathfrak {g}_\infty ,K_\infty ,\Pi \otimes E_\mu )\rightarrow H^{b_n}(\mathfrak {g}_\infty ,K_\infty ,{}^\sigma \Pi \otimes {}^\sigma E_\mu )$.

With respect to these two rational structures on relative Lie algebra cohomology and the $\sigma$-linear bijections $\tilde{\sigma }$, the proof of Prop. 3.1 goes through word-for-word, recalling the validity of [17, Theorem (5.1)] for $\Pi '_v$, $v\notin S_\infty$. Hence, we obtain this way Whittaker-periods $p(\Pi )$ and $p(\Pi ')$, well-defined up to multiplication by $\mathbb {Q}(\Pi _f)^*$, resp. $\mathbb {Q}(\Pi '_f)^*$. In turn, again as in Prop. 3.1, these periods define rationally normalized isomorphism $\Theta ^\mathsf{cusp}_0$ and $\Theta ^\mathsf{Eis}_0$ of the corresponding Whittaker models and relative Lie algebra cohomologies.

### Statement and proof of the main theorem

#### Theorem 1.9

Let *F* be any CM-field. Let $\Pi$ be a cuspidal automorphic representation of $\mathrm{GL}_n(\mathbb {A})$ (as in Sect. 1.2.4) which is cohomological with respect to $E_\mu$ and let $\Pi '$ by an isobaric automorphic representation of $\mathrm{GL}_{n-1}(\mathbb {A})$ (as in Sect. 1.2.5) which is cohomological with respect to $E_\lambda$ and of central character $\omega _{\Pi '}$. We assume that the highest weights $\mu =(\mu _v)_{v\in S_\infty }$ and $\lambda =(\lambda _v)_{v\in S_\infty }$ satisfy the interlacing-hypothesis 1.5. Then the following holds:

1. For all critical values $\tfrac{1}{2}+m\in {\mathrm{Crit}}(\Pi \times \Pi ')$ and every $\sigma \in \mathrm{Aut}(\mathbb {C})$,
  $$\begin{aligned}&\sigma \left( \frac{L\left( \tfrac{1}{2}+m,\Pi _f \times \Pi '_f\right) }{p(\Pi )\ p(\Pi ')\ p(m,\Pi _\infty ,\Pi '_\infty ) \ \mathcal {G}(\omega _{\Pi '_{f}})}\right) \\&\quad = \frac{L\left( \tfrac{1}{2}+m,{}^\sigma \Pi _f \times {}^\sigma \Pi '_f\right) }{p({}^\sigma \Pi )\ p({}^\sigma \Pi ')\ p(m,{}^\sigma \Pi _\infty ,{}^\sigma \Pi '_\infty ) \ \mathcal {G}(\omega _{{}^\sigma \Pi '_{f}})}. \end{aligned}$$
2. $$\begin{aligned} L\left( \tfrac{1}{2}+m,\Pi _f \times \Pi '_f\right) \ \sim _{\mathbb {Q}(\Pi _f)\mathbb {Q}(\Pi '_f)} p(\Pi )\ p(\Pi ') \ p(m,\Pi _\infty ,\Pi '_\infty ) \ \mathcal {G}(\omega _{\Pi '_{f}}), \end{aligned}$$
  where “$\sim _{\mathbb {Q}(\Pi _f)\mathbb {Q}(\Pi '_f)}$” means up to multiplication by an element in the composition of number fields $\mathbb {Q}(\Pi _f)\mathbb {Q}(\Pi '_f)$.

#### Proof

As a first step, we observe that Lemma 3.4 and the results of §3.8 transfer verbatim from [12] to our case here. Hence, recollecting all the preparatory results of this note, the following diagram, which amplifies the main diagram of §3.2, is finally well-defined:As a next step, we observe that the results of [17, 18], as well as [6, Lemme 4.6 ] are valid for $\Pi _v$, whenever $\psi =\otimes _v\psi _v$ is unramified at $v\notin S_\infty$, whence the proof of [23, Prop. 2.3.(c)] carries over to the situation considered here. In other words, the correction-factors $c_{\Pi _v}$ of Sect. 1.4.1 satisfy $\sigma (c_{\Pi _v})= c_{{}^\sigma \Pi _v}$ for all $\sigma \in$ $\hbox {Aut}(\mathbb {C})$ and at all non-archimedean places, where both $\Pi '$ and $\psi$ are unramified.

As a final consequence, the proof of [12, Thm. 3.9] now goes through word-for-word in our more general situation at hand and we hence obtain Theorem 1.9 (1) by chasing our special Whittaker vectors $\xi _{\Pi _f}:=\otimes _{v\notin S_\infty }\xi _{\Pi _v}$ and $\xi _{\Pi '_f}:=\otimes _{v\notin S_\infty }\xi _{\Pi '_v}$ through the above diagram. Assertion (2) follows from (1) applying Strong Multiplicity One for isobaric automorphic representations ([19], Thm. 4.4) together with Multiplicity One ([10] §3.3 and [16, 30]). $\square$

#### Remark 1.10

Theorem 1.9 represents a rather vast generalization of [26, Thm. 1.1] and [25, Thm. 1.1] over general CM-fields *F*: In the latter references, the analogous result has been shown for cuspidal automorphic representations $\Pi '$ (over $F=\mathbb {Q}$ in [26] and over a general number field *F* in [25])—a condition, which we stretched to all isobaric sums $\Pi '$, which are fully-induced from cuspidal representation $\Pi _1, \ldots , \Pi _k$ (as in Sect. 1.2.5) over arbitrary CM-fields *F*. The situation for isobaric representations over general number fields *F* will be significantly more complicated, notably at infinity.

## A consequence

### Ratios of critical values

The following result is a direct consequence of our main result. It avoids any reference to Whittaker periods and expresses quotients of critical values of $L(s,\Pi \times \Pi ')$ in terms of archimedean factors only. The reader may compare this corollary to the main result of [15] on quotients of consecutive critical values of Rankin–Selberg *L*-functions attached to cuspidal representations $\Pi$ and $\Pi '$ over totally real fields.

#### Corollary 2.1

Let *F* be any CM-field. Let $\Pi$ be a cuspidal automorphic representation of $\mathrm{GL}_n(\mathbb {A})$ (as in Sect. 1.2.4) which is cohomological with respect to $E_\mu$ and let $\Pi '=\Pi _1\boxplus \cdots \boxplus \Pi _k$ by an isobaric automorphic representation of $\mathrm{GL}_{n-1}(\mathbb {A})$ (as in Sect. 1.2.5) which is cohomological with respect to $E_\lambda$ and of central character $\omega _{\Pi '}$. We assume that the highest weights $\mu =(\mu _v)_{v\in S_\infty }$ and $\lambda =(\lambda _v)_{v\in S_\infty }$ satisfy the interlacing-hypothesis 1.5. Let $\tfrac{1}{2}+m,\tfrac{1}{2}+\ell \in \text {Crit }(\Pi \times \Pi ')$ be two critical values and abbreviate $\Omega _{\Pi _\infty ,\Pi '_\infty }(m,\ell ):=p(m,\Pi _\infty ,\Pi '_\infty ) p(\ell ,\Pi _\infty ,\Pi '_\infty )^{-1}$. Then, whenever $L^S(\tfrac{1}{2}+\ell , \Pi \times \Pi ')$ is non-zero (e.g., if $\Pi$ is unitary and $\ell \ne 0$),

$$\begin{aligned} \frac{L^S\left( \tfrac{1}{2}+m, \Pi \times \Pi '\right) }{L^S\left( \tfrac{1}{2}+\ell , \Pi \times \Pi '\right) } \sim _{\mathbb {Q}(\Pi _f)\mathbb {Q}(\Pi '_f)} \Omega _{\Pi _\infty ,\Pi '_\infty }(m,\ell ), \end{aligned}$$

and hence only depends on the archimedean components $\Pi _\infty$ and $\Pi '_\infty$.

In particular, if $L^S(\tfrac{3}{2}+m, \Pi \times \Pi ')$ is non-zero (e.g., if $\Pi$ is unitary and $m\ne -1$), then the quotient of consecutive critical *L*-values satisfies

$$\begin{aligned} \frac{1}{\Omega _{\Pi _\infty ,\Pi '_\infty }(m,m+1)}\frac{L^S\left( \tfrac{1}{2}+m, \Pi \times \Pi '\right) }{L^S\left( \tfrac{3}{2}+m, \Pi \times \Pi '\right) } \in \mathbb {Q}(\Pi _f)\mathbb {Q}(\Pi '_f). \end{aligned}$$
